# Data integration of 104 studies related with microRNA epigenetics revealed that miR-34 gene family is silenced by DNA methylation in the highest number of cancer types

**DOI:** 10.15190/d.2014.10

**Published:** 2014-06-30

**Authors:** Ziga Strmsek, Tanja Kunej

**Affiliations:** Department of Animal Science, Biotechnical Faculty, University of Ljubljana, Groblje 3, 1230, Domzale, Slovenia

**Keywords:** microRNA, miRNA, miR-34, DNA methylation, cancer, Tanja, Kunej, University of Ljubljana, Slovenia

## Abstract

There is an increasing research interest regarding deregulation of microRNA (miRNA) expression by DNA methylation in cancer. The aim of this study was to integrate data from publications and identify miRNA genes shown to be silenced in the highest number of cancer types and thus facilitate biomarker and therapeutic development. We integrated relevant data from 104 published scientific articles. The following databases and bioinformatics tools were used for the analysis: miRBase, miRNA Genomic Viewer, MultAlin, miRNA SNiPer, TargetScan, Ensembl, MethPrimer, TarBase, miRecords, and ChIPBase. Among 2578 currently known human miRNAs and 158 known to be regulated by DNA methylation, miR-34 gene family (miR-34a, -34b, and -34c) was shown to be silenced by DNA methylation in the highest number of cancer types. Consequently, we developed the miR-34 gene family regulatory atlas, consisting of its upstream regulators and downstream targets including transcription factor binding sites (TFBSs), CpG islands, genetic variability and overlapping QTL. MicroRNA-34 gene family has a potential as a cancer biomarker and target for epigenetic drugs. This potential has already been recognized as MRX34 is well into phase I studies. The developed miR-34 gene family regulatory atlas presented in this study provides a starting point for further analyses and could thus facilitate development of therapeutics.

## INTRODUCTION

MicroRNAs (miRNAs) are small non-coding RNAs, 19 – 25 nucleotides in length that regulate the translation and degradation of target mRNAs, and control approximately 60% of human genes^1^. By binding to the different target gene regions, *i.e.,* 3'-untranslated region (3'-UTR), 5'-UTR, promoter, or coding sequences, they repress or activate translation (reviewed in^2^). The role of miRNA genes in cancer have been discovered early after their discovery because 1.) they are located on breaking-prone and cancer-associated genomic regions^3^, 2.) they are involved in regulation of cell proliferation and apoptosis^4,5^ and 3.) they were found to be deregulated in malignant tumors and tumor cell lines (oncomiR) in comparison with normal tissues^6-8^. MicroRNA genes can represent two opposing roles, either behaving as oncogenes or tumor suppressors (eg. miR-34 gene family) depending on the tissue type and presence of specific targets^9^. Importance of miRNAs in cancer has already been recognized as miRNA based drug MRX34 has already entered phase I studies^10^. Aberrant miRNA gene expression signatures (either up- or down-regulated) are characteristic in cancer cells^11^. MicroRNA genes are regulated on various levels including epigenetic silencing with histone modification and/or DNA methylation of CpG islands that encompass or are located adjacent to miRNA genes. Moreover, the frequency of miRNA gene methylation and consequently the frequency of epigenetic regulation are at about one order of magnitude higher than that of the protein-encoding genes^12,13^.

A lot of scientific articles independently reported aberrant DNA methylation of various miRNA genes in numerous cancer types. Most of these studies reported hypermethylation of upstream regions, thus resulting in downregulation of miRNA genes. Integrative review by Kunej et al.^12^, that included data from 45 studies published till 7/2010, revealed that miRNAs can be regulated by DNA methylation in only one cancer type, thus indicating that they are cancer type specific. On the other hand, miRNA can be silenced by DNA methylation in various cancer types, thus offering general cancer biomarkers. For example, miR-34a was reported to be silenced by DNA methylation in 12 cancer types^12^. Additionally, majority of scientific articles also used miR-127 as an experimental internal control in epigenetic studies of miRNA genes since it was the first miRNA shown to be epigenetically regulated in several cancer types^14^.

In this study we present further integration of data from publications published till 5/2014 which revealed that miR-34 gene family is regulated by DNA methylation in the highest number of cancer types – consequently this gene family was the focus of the current study. The results indicated that miR-34 family could be used as general cancer biomarker. However, the number of publications related to this topic is increasing and there is no systematic integration of publications and no clear data regarding biomarker and cure potential of the miR-34 gene family. Therefore, the aim of this study was to: 1.) develop the catalog of cancer types associated with miR-34 gene family silencing by DNA methylation, 2.) create the miR-34 gene family regulatory atlas and 3.) discuss miR-34 gene family’s therapeutic potential and thus facilitate biomarker and therapeutic development.

## MATERIALS AND METHODS

We reviewed literature published from 2006 to 5/2014 searching for publications through PubMed using key phrases: microRNA (miRNA), DNA methylation, CpG island, cancer, oncogene, epigenetics. Genomic locations and sequences of miR-34 gene family were obtained from miRBase^15^, release 20, June 2013. Visualization of genomic location of miRNA and quantitative trait locus (QTL) was performed using miRNA Genomic Viewer (http://www.integratomics-time.com/ miRNA-genomic-viewer/). Sequences of 2 kb upstream of miR-34 family genes and single-nucleotide polymorphisms (SNPs) were obtained from Ensembl (release 70 (1/2013)). CpG island analysis was performed using MethPrimer^16^ tool using the default parameters: window size >100, observed/expected >0.6, GC percent >50 %). Mature miRNA seed regions and SNPs were determined using miRNA SNiPer 3.0^17,18^and TargetScan^19^ (release 6.2). Analysis of transcription factor binding sites (TFBSs) was performed using ChIPBase^20^ (parameters: hg19, all transcription factors, 5 kb upstream). Experimentally validated targets were obtained from TarBase^21^ (version 6.0) and miRecords^22^ databases and published literature. Alignments of pre-miRNA sequences were performed using MultAlin tool^23^.

## RESULTS 

In this study we performed literature mining to determine miRNAs that were most frequently showed to be deregulated by epigenetic mechanisms therefore having the potential for general cancer biomarkers and therapeutic targets. The results revealed that from all 2578 currently known human mature miRNA genes, miRNA gene family miR-34 has been found to be regulated by DNA methylation in the highest number of cancer types. Consequently, we focused the research on miR-34 gene family and developed the catalog of cancer types which were shown to be associated with regulation of miR-34 gene family by DNA methylation that is available online. Additionally, we also developed the miR-34 gene family regulatory atlas, consisting of its upstream regulators, overlapping genomic elements and downstream targets (transcription factor binding sites (TFBSs), CpG islands, genetic variability, and QTL). Moreover, we discussed potential of miR-34 gene family as biomarker, cure and epigenetic experimental control.

### Catalog of cancer types associated with miR-34 silencing by DNA methylation

**Figure 1:**
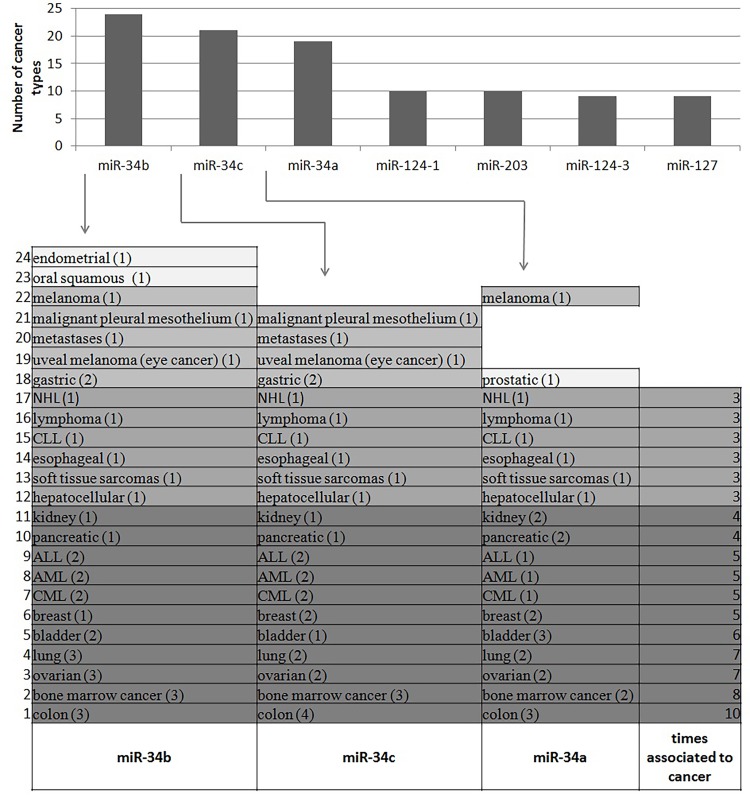
MicroRNA genes shown to be regulated by DNA methylation in more than nine cancer types The number in bracket represents the number of reports describing the association between miRNA gene that is silenced by DNA methylation and cancer type. The darker the colour, the stronger association between miRNA gene and cancer type. ALL (acute lymphoblastic leukemia), AML (acute myeloid leukemia), CML (chronic myelogenous leukemia), CLL (chronic lymphocytic leukemia), NHL (non-Hodgkin lymphoma). Bone marrow cancer consists of myeloproliferative neoplasms, myeloma and lymphoma.

We performed an extensive literature mining and collected of 104 papers related with epigenetic silencing of miRNA genes by DNA methylation in cancer. Data integration revealed 158 miRNA genes that have been shown to be deregulated by DNA methylation in 34 different cancer types. Analysis revealed that miR-34 gene family is regulated by DNA methylation in the highest number of cancer types (**Figure 1**), thus confirming results from our previous integrative study^12^. Out of 104 papers 28 studies described aberrant DNA methylation of miR-34 gene family in 25 cancer types^24-52^. Out of these 28 studies, five were performed at the genome-wide or multi-loci level using microarrays32 and quantitative reverse transcription PCR (RT-PCR)^27,42,43,49^, the remaining 23 papers focused on the members of miR-34 family. **Figure 1** represents the number of different cancer types that were reported to be associated with miR-34 gene silencing by DNA methylation. MicroRNA-34b was found to be silenced by DNA methylation in 24, miR-34c in 21 and miR-34a in 19 cancer types. All three members of miR-34 gene family were silenced by DNA methylation in 17 cancer types. MicroRNA-34b and miR-34c were additionally silenced by DNA methylation in a set of four cancer types. Moreover miR-34b and miR-34a were silenced by DNA methylation in melanoma. Furthermore miR-34b had been silenced by DNA methylation in oral squamous and endometrial cancer while miR-34a had been silenced by DNA methylation in prostatic cancer. Colon cancer was most frequently associated with epigenetic silencing of miR-34 gene family; five studies described ten associations between the miR-34 gene family members and colon cancer^24,27,34,40,51^. Other cancer types have also been frequently linked with miR-34 gene family: bone marrow cancer eight times, ovarian and lung cancer seven times, bladder cancer six times, breast, chronic myelogenous leukemia (CML), acute myeloid leukemia (AML), acute lymphoblastic leukemia (ALL) five times and pancreatic and kidney cancer four times. Moreover, some papers reported that beside DNA methylation histone modifications are additional epigenetic regulators of miR-34 gene family expression^32,42^. Detailed catalog of cancer types which were shown to be associated with regulation of miR-34 gene family by DNA methylation is available online (http://www.integratomics-time.com/epigenetics/ catalog2).

### MicroRNA-34 gene family regulatory atlas

**Figure 2:**
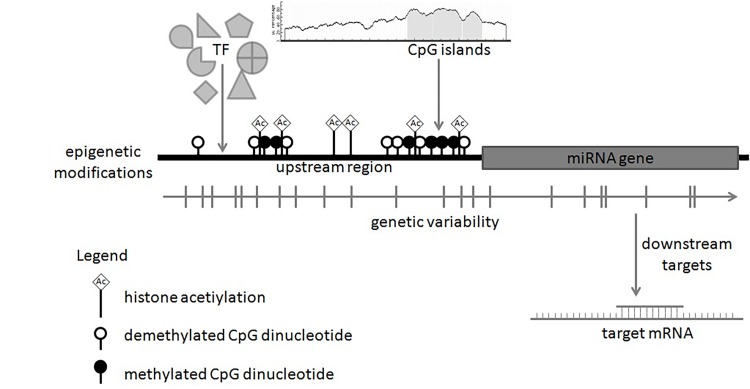
A miR-34 gene family regulatory atlas An atlas integrates relevant genomic data regarding DNA methylation, histone modifications, CpG islands, upstream regulators, downstream targets and genetic variability.

To gain the insight into a miR-34 gene regulation we developed a miR-34 gene family regulatory atlas by integrating the following data: 1.upstream regulators (TFBSs, CpG islands), 2.overlapping genetic elements (genetic variability, QTL), and 3. experimentally validated downstream targets (**Figure 2**).

**Figure 3:**
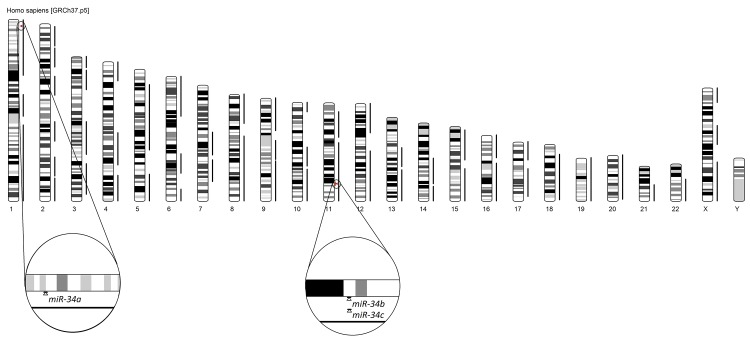
Genomic location of miR-34-a, -b, and –c genes and overlapping QTL Analysis was performed using miRNA genomic viewer and revealed that all three members of the miR-34 family genes overlapped with QTL for prostatic neoplasms

MicroRNA-34 gene family maps to two chromosomes: 1p36.22 (miR-34a) and 11q23.1 (miR-34b and miR-34c) (**Figure 3**). The analysis using the miRNA Genomic Viewer tool revealed that all three miR-34 gene family genes overlap with QTL for prostatic neoplasms and that miR-34b/c overlap with QTL associated with breast neoplasms.

**Figure 4:**
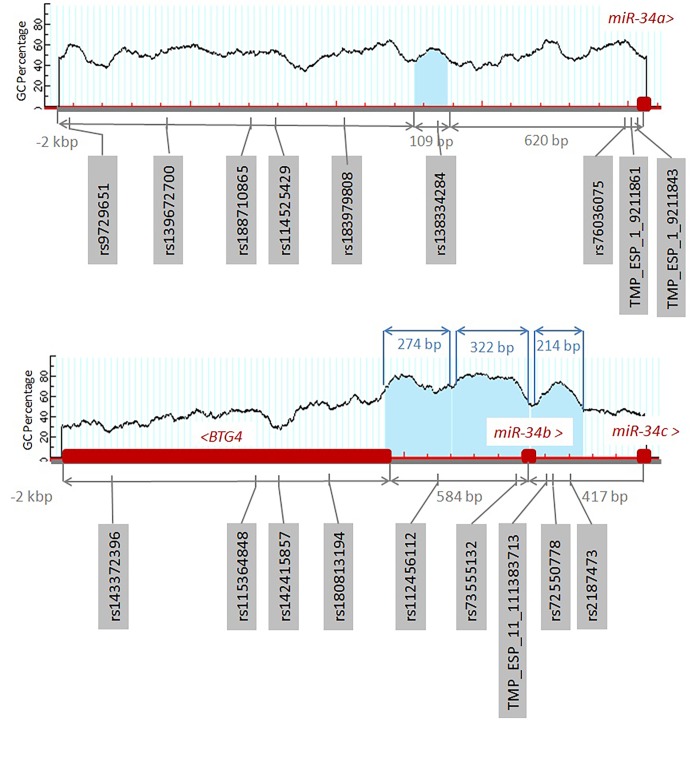
CpG island analysis of miR-34 family and SNPs that could affect gain/loss of DNA methylation sites One CpG island is located upstream of the miR-34a gene. Three CpG islands are located upstream of the miR-34b/c gene region and miR-34b is embedded in the CpG island. Promoter for miR-34b/c is birectional and it also regulates BTG4. Six SNPs overlap CpG dinucleotides within promoter regions: one SNP is located upstream of miR-34a gene and five upstream of the miR-34b/c gene.

MicroRNA genes miR-34b and miR-34c are located 494 bp from one another. In silico CpG analysis revealed that both miR-34a and miR-34b/c genes are associated with one and three predicted CpG islands respectively (**Figure 4**). There is one CpG island within the 2 kb upstream region of miR-34a; located 620 bp from the beginning of miR-34a gene and comprises 109 bp. Three CpG islands are located within 2 kb upstream region of miR-34b/c gene. MicroRNA-34b is imbedded in the second and third CpG island. Moreover, promoter region of miR-34b/c gene is birectional and also drives expression of BTG4^27^.

Inquiry using miRNA SNiPer tool showed that pre-miR-34a is polymorphic, as it has one SNP in the seed region of miR-34a-5p and one in the loop of pre-miR-34a^17^. Moreover, the analysis of the SNPs, deposited in the Ensembl database showed that SNPs within the upstream region of the miR-34 overlapped with CpG dinucleotides and could therefore potentially cause gain or loss of the methylated sites. In the upstream 2 kb region there are nine and nine SNPs in each; miR-34a and miR-34b/c gene, which are located within CpG dinucleotides and could cause gain or loss of the CpG dinucleotides, including one and five SNPs that are located within predicted CpG islands, respectively.

Additionally, our query at ChIPBase revealed, that there are four common TFBSs present within the 5 kb upstream regions of the miR-34 gene family (CDX2, FoxA1, HNF4A, and p63), while miR-34a has additional 18 TFBSs within the 5kb upstream region. Moreover, miR-34 gene family is a downstream target of p53^32,53^. Comparison of ChIPBase data and genetic variability from Ensembl revealed that within *miR-34a* upstream region two SNPs reside within two TFBSs: transcription factor HNF4A binds to -24 bp (MP_ESP_1_9211861; C>T) and TAF1 binds to -91 bp (rs116877979; A>C).

**Figure 5:**
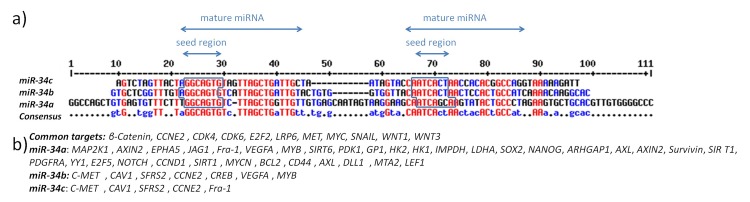
Alignment of the miR-34 family pre-miRNA sequences and experimentally validated targets of the miR-34 gene family a) Alignment of pre-miRNA sequences of miR-34 gene family. Red nucleotides are present in all three members of miR-34 gene family, blue nucleotides are present in two members of miR-34 gene family. Seed regions are represented in squares. b) Experimentally validated targets of miR-34 family genes.

To collect all known data regarding downstream targets of miR-34 gene family we integrated data from databases specialized for collecting of experimentally validated miRNA targets (TarBase, miRecords) and the literature^24,27,28,30,34,50,54,55^. The data collection revealed that miR-34 family, which has conserved seed regions has several experimentally validated targets (**Figure 5**): miR-34a, b, and c have 45, 19 and 16 experimentally validated targets respectively. There are 11 mRNAs targeted by all three members of miR-34 gene family, four mRNAs that are targeted by miR-34b and miR-34c, two mRNAs that are targeted by miR-34a and miR-34b and one mRNA that is targeted by miR-34a and miR-34c. For example, Toyota et al. reported that MET, CCNE2, CDK4, CAV1, MYB, SFRS2 mRNAs are targeted by miR-34b/c in colon cancer cell lines and tumors^27^.

To analyze if there are other miRNAs which could vicariate the function of miR-34 family, we performed a comparison of all known miRNA seed regions, which are responsible for target binding. Comparison of seed regions between miR-34 family with all known miRNAs revealed that miR-449a and miR-449b have the same seed sequence as miR-34a and miR-34c (GGCAGUG) and miR-449c has the same seed sequence as miR-34b (AGGCAGU).

The developed miR-34 gene family regulatory atlas and catalog of cancer types which were shown to be associated with regulation of miR-34 gene family by DNA methylation will further improve our understanding of role of miR-34 gene family in cancer.

## DISCUSSION

### Catalog of cancer types associated with miR-34 silencing by DNA methylation

Out of all 75 possible combinations of miR-34 family genes (n=3) and 25 analyzed cancer types, scientific papers reported 64 combinations of miR-34 family genes and cancer types. Out of these 64 combinations, 26 (40.6%) have been reported in more than one study thus enabling more reliable confirmation for epigenetic silencing. MicroRNA gene family miR-34 has a potential to be used as general cancer biomarker or as experimental internal control because combination of *miR-34a*, *miR-34b* and *miR-34c* was silenced by DNA methylation in 25 out of all 35 (71.4%) analyzed cancer types. The majority of researchers used miR-127 as internal experimental control as it was the first discovered miRNA to be epigenetically regulated^14^. However, our analysis revealed that miR-127 was deregulated by DNA methylation in nine cancer types, while miR-34 gene family was silenced by DNA methylation in 25 cancer types; thus we are proposing that in the future experiments miR-34 family could be also used as control.**

**Figure 6:**
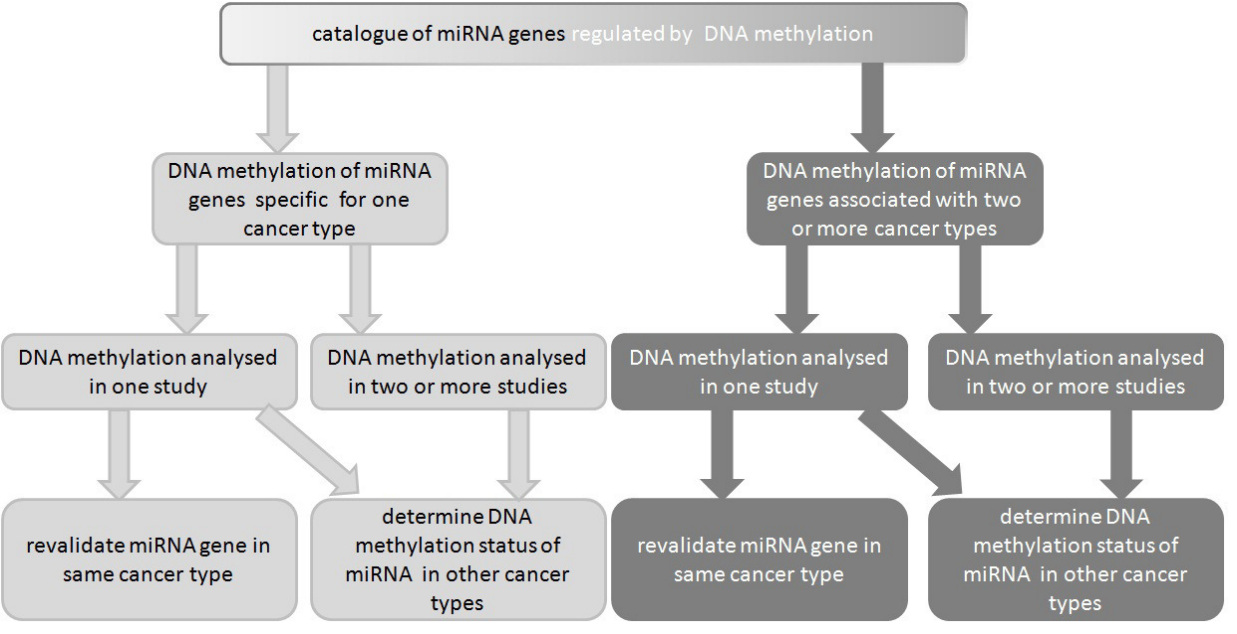
Proposed decision tree for identification of miRNA genes silenced by DNA methylation in cancer

It is very likely that with the upcoming publications the number of cancer types with deregulated miRNA genes by DNA methylation will increase thus revealing which miRNA is more suitable as gold standard. Furthermore, the focus of the studies is not evenly distributed across cancer types; colon cancer is one of the most researched cancer types, while oral squamous, prostatic and endometrial cancer have been researched in only one publication. Thus, we are proposing a decision tree for a more efficient planning of the future experiments by the interested scientific community (**Figure 6**).

### MicroRNA-34 gene family regulatory atlas

According to previous research^12^, 20% of miRNA genes regulated by DNA methylation in cancer had CpG islands in 5 kb upstream region and among them 14% of miRNA genes had CpG islands that overlap with miRNA genes and *miR-34b/c* gene being one of them. It has been previously shown that *miR-34b/c* gene is regulated by a birectional promoter, which is shared with the *BTG4* gene^27^. Moreover, the promoter activity in the *miR-34b/c* direction was almost twice that in the *BGT4* direction. Furthermore BGT4 also functions as tumor suppressor as it has anti-proliferative properties and can induce G1 cell cycle arrest. Also, miR-34 gene family was reported to be direct p53 target, which induces apoptosis, senescence and cell cycle arrest (reviewed in^32^). Welch et al.^56^ reports that 1q36.22 is frequently deleted in neuroblastoma thus causing down regulation of miR-34a without epigenetic mechanisms. Moreover, there are SNPs present in the CpG islands upstream of miR-34 gene family genes, some of them might affect gain or loss of the CG dinucleotides thus resulting in another potential source of aberrant methylation thus having biomarker potential. An example of miRNA promoter annotation is presented in Baer et al.^57^ where they discuss the problem of promoter annotation using gene family miR-34 as an example.

As members of miR-34 gene family have conserved seed sequences (**Figure 5** [a]), some targets are common for all three members of the miR-34 gene family (**Figure 5** [b]); the analysis revealed that 11 mRNAs (*MYC, CDK6, E2F3, CDK4, LRP6, WNT3, WNT1, SNAIL, MET, CCNE2 *and *β-Catenin*) are targeted by all three members of miR-34 family. There are also seven genes that are targeted by two of the miR-34 family – *C-MET, CAV1, SFRS2, CCNE2* are targeted by both miR-34b and miR-34c, while *VEGFA* and *MYB *are targeted by miR-34a and miR-34b and *Fra-1* is targeted by both miR-34a and miR-34c. Moreover, recent study indicates that inhibition of *Myc* in mice kills lung cancer cells^58^, thus making the opportunity to regulate *MYC* expression with miR-34 family effective and efficient cure for cancer. Similar experiment also identified miR-34a as a potential inhibitor of prostate cancer cells and metastasis by targeting CD44^59^.

Comparison of miRNA seed regions revealed that other miRNA have the same seed sequence as members of miR-34 gene family. Moreover, it has been previously shown that a SNP within seed region could cause the formation of the seed region annotated to a different miRNA^17,18^; therefore there is a possibility that SNPs within seed regions of other miRNA could cause formation of seed region annotated to member of gene family miR-34. However, to our knowledge to date there are no reports of SNPs that would cause formation of miR-34 gene family seed regions. Moreover, SNPs that are located within the transcription factor binding sequence of *miR-34a* could have potential therapeutic value as they could affect expression of miR-34a by gain or loss of transcription binding sites; however, the effects of these SNPs on expression of miR-34a have yet to be experimentally tested. The developed atlas could now be complemented with novel discovered regulatory elements.

### Cure and biomarker potential

Reexpression or ectopic expression of one of the members of miR-34 gene family led to inhibition of cellular proliferation and enhancement of apoptosis in myeloma cells^44^, suppression of migration and invasion in prostate cancer cell lines^60^ and reduction in cell invasion, motility and attachment rates in the stage 3 and stage 4 melanoma cell lines^43^. These studies indicate that miR-34 gene family plays an important role in cancer development, invasion and survivability. This combined data suggests that azacitidine (Aza-dC) and other demethylating agents (methyltransferase inhibitors) would be useful treatment for solid tumors. However, as demethylating agents are not specific and they demethylate DNA randomly, their side effect may be severe. However, a miR-34 gene family specific demethylating drug or ectopic expression of miR-34 gene family would offer effective cure for cancer with little to no side effects. This concept has already been proven in study^61^ in which transplanted tumors in mice were treated with miR-34a mimics and complete inhibition of tumor growth was achieved without any detectable side effects. However, as cancer tissue is composed of many independent subclones this experiment does not fully represent in vivo conditions and effect, but it serves as a proof of concept that miR-34 gene family has cure potential that has to be researched and proven extensively in the future studies. First breakthrough has already been achieved as a drug with MRX34 entered Stage I in April 2013 (reviewed in^55^). Moreover the potential of miRNA and long non-coding RNA (lncRNA) for treatment of cancer has already been recognized and is the topic of increasing research (discussed in^62^).**

Specific biomarker potential for miR-34 gene family has already been recognized in other scientific papers, but no scientific paper reports miR-34 gene family as general cancer biomarker. A lot of scientific papers are proposing that members of miR-34 gene family could be used as biomarker for specific cancer type - but we are proposing miR-34 gene family should be used as a general cancer biomarker, as it has been found to be silenced by DNA methylation in 25 cancer types^37,42^. Moreover, miR-34 gene family’s DNA methylation was found to be cancer cells specific, as normal tissue has demethylated CpG islands, while cancer cells have aberrant CpG DNA methylation^27-30,34,50,54,55^. As biomarker and therapeutic development is multistage and highly complex procedure it can be aided and facilitated by integrative projects such as Integratomics TIME (http://www.integratomics-time.com/home).

## CONCLUSIONS

In conclusion, our systematic data integration and bioinformatics analysis revealed that miR-34 family could be used as a general cancer biomarker because miR-34 family is silenced by DNA methylation in a high number of cancer types. Furthermore, our and other studies conducted on the topic of epigenetic regulation of miR-34 gene family indicate that miR-34 gene family is a master regulator of tumor biology. Moreover, in comparison to protein-coding genes miRNA offer better biomarker properties as the frequency of miRNA gene DNA methylation is an order of magnitude higher. We are also proposing that the researchers could use miR-34 gene family as experimental internal control in epigenetic studies. But still more preclinical studies related with miR-34 gene family need to be conducted in order to better characterize its regulation and its downstream molecular pathways. Furthermore, researchers should focus on combinations of miR-34 family genes and cancer types that have not already been proven to be silenced by DNA methylation as proposed by decision tree. Therefore, the supplemented catalog available at http://www.integratomics-time.com/epigenetics/ catalog2 and the miR-34 gene family regulatory atlas presented in this study provide a starting point for further analyses and could thus facilitate development of therapeutics.

**♦** **MicroRNA-34 gene family is regulated by DNA methylation in the highest number of analyzed cancer types.**

**♦** **MicroRNA-34 gene family regulatory atlas integrates relevant data regarding regulation of miR-34 gene family, up- and down-stream targets, single-nucleotide polymorphisms (SNPs), CpG islands and could thus serve as a foundation for future functional research.**

**♦ DNA methylation status of miR-34 gene family has not been determined in all cancer types - thus we are proposing a decision tree, based on which future research could be more efficiently organized. MicroRNA mimetics are now in clinical trials for a wide variety of malignancies.**
